# Relapsing Polychondritis in a Patient With Rheumatoid Arthritis: Improved With Pentoxifylline

**DOI:** 10.7759/cureus.48849

**Published:** 2023-11-15

**Authors:** Kiana Banafshay, Blayne Fenner, Kristina Blegen, Jackson Driskill, Michelle Tarbox

**Affiliations:** 1 Dermatology, Texas Tech University Health Sciences Center School of Medicine, Lubbock, USA; 2 Dermatology, Texas Tech University Health Sciences Center, Lubbock, USA

**Keywords:** pentoxifylline, vexas, cartilaginous tissue, systemic autoimmune disease, rheumatoid arthritis, auricular chondritis, relapsing polychondritis

## Abstract

Relapsing polychondritis (RP) is a rare autoimmune condition that involves the recurrent inflammation of cartilage throughout the body, with a predilection for auricular and nasal cartilage. Given its rarity and diverse clinical presentations, RP is frequently misdiagnosed or left untreated, which can lead to significant morbidity and mortality. When it is correctly diagnosed, there are no standardized guidelines on the treatment of RP to date. Management of this disease requires a multidisciplinary approach, and about 30% of patients with RP have other autoimmune disorders, further complicating the approach to targeted treatment. Biologic agents (including TNF inhibitors) are commonly used. We present a compelling case of a 46-year-old female with rheumatoid arthritis (well-controlled on adalimumab) and hypothyroidism who presented to the dermatology clinic with recurrent episodes of painful, swollen, and erythematous ears, leading to a clinical diagnosis of relapsing polychondritis. Off-label use of oral pentoxifylline, along with topical corticosteroids, led to significant improvement in her symptoms. Dermatologists play an important role in the diagnosis of this rare disorder, as skin manifestations may be the initial presenting sign of RP. Further research into potentially effective treatments is needed. Timely identification and management of RP may prevent the progression of cartilage destruction, thus improving patients’ long-term prognosis and overall quality of life.

## Introduction

Relapsing polychondritis (RP) is a rare autoimmune condition with recurrent inflammation in cartilage throughout the body, often targeting the ears, nose, peripheral joints, and tracheobronchial tree [1]. The prevalence of RP is 3.5 out of 1,000,000 Americans, equally affecting races and sex. Approximately 30% of patients with RP have an associated autoimmune disorder, rheumatological disease, or vasculitis, and there are also reported associations with malignancy [2]. Dermatologic manifestations may be the initial presenting sign of RP, highlighting the dermatologist’s role in early recognition of this disease and possibly preventing later complications [3]. However, given the rarity of RP, it is frequently misdiagnosed or untreated. Even when RP is correctly diagnosed, treatment can be challenging, as current treatment standards are empirical and primarily based on published case reports. However, the existing literature offers biologics as one modality of treatment. We present a case of refractory RP despite the concurrent use of adalimumab, which subsequently improved with the addition of pentoxifylline and topical corticosteroids.

## Case presentation

**Figure 1:**
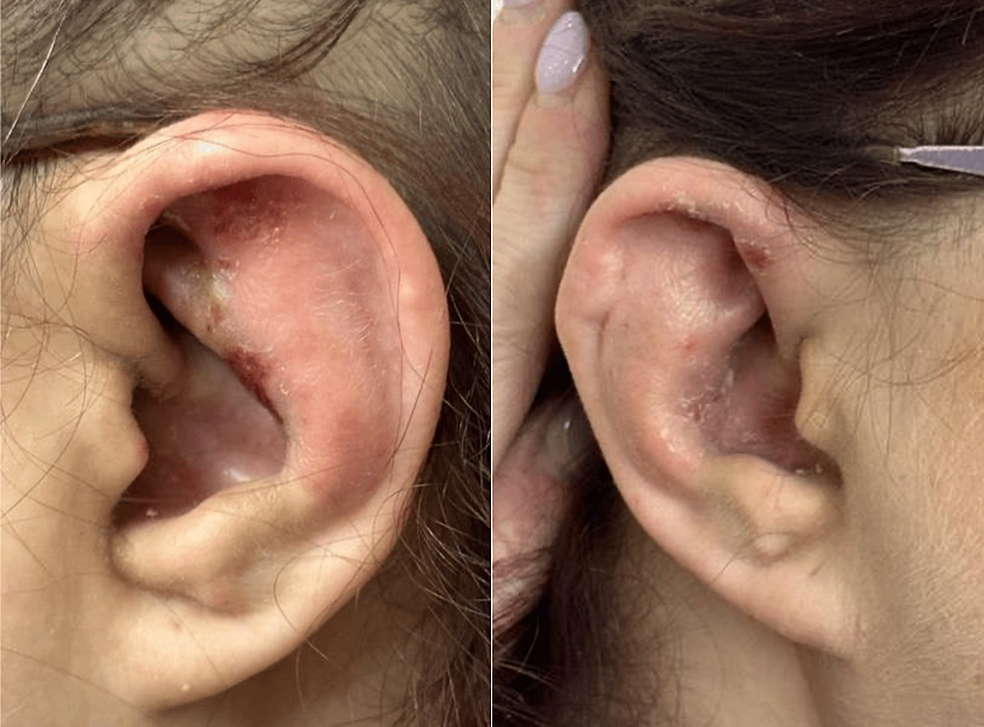
Erythema and swelling of the bilateral auricles in our patient at initial presentation. Focal areas of superficial heme crust and a “cauliflower” appearance to the ears are also seen.

A 46-year-old female with a past medical history of rheumatoid arthritis and hypothyroidism presented to the dermatology clinic with refractory episodes of painful, swollen, and erythematous ears. A review of systems revealed intermittent dyspnea, arthralgias, and rhinorrhea. Her home medications included adalimumab, diclofenac, and levothyroxine. The patient has been well-controlled for several years on adalimumab for her rheumatoid arthritis. Physical examination was notable for erythema, swelling, and exquisite tenderness to the auricles of both ears, with sparing of the lobules (Figure 1). Focal areas of superficial heme crust and a “cauliflower” appearance to the ears correlated with the acute-on-chronic nature of her symptoms. Based on her history and exam findings, a clinical diagnosis of relapsing polychondritis was strongly considered and made.

Subsequent diagnostic tests included an unremarkable chest X-ray and labs notable for mild leukocytosis and mildly elevated alanine aminotransferase (ALT). Erythrocyte sedimentation rate (ESR), antinuclear antibody (ANA), immunoglobulin G (IgG) subclass 4, thyroid function, and Quantiferon gold tests were all within normal limits. The patient was experiencing adequate control of her rheumatoid arthritis on adalimumab; therefore, the decision was made to treat her RP with oral pentoxifylline 400 mg TID (off-label use) and topical clobetasol 0.05% ointment daily. Pentoxifylline was chosen for several reasons, including its anti-inflammatory properties, low expense, and tolerability. Additionally, since the patient was already on adalimumab, we did not want to start the patient on another systemic immunosuppressant. We did consider dapsone as another potential treatment option; nevertheless, due to the necessity of frequent lab monitoring and the patient's financial constraints, we decided against it. At follow-up one month later, the patient reported improved pain and appearance of her ear lesions, with no subsequent flares of auricular chondritis within that time frame. She reported this improvement had been maintained for six months after starting therapy.

## Discussion

Relapsing polychondritis is a rare immune-mediated connective tissue disorder characterized by recurrent episodes of cartilaginous inflammation, with subsequent progressive anatomical deformities and functional impairment of the involved structures [1]. While the exact pathogenesis is unknown, circulating autoantibodies against collagens II, IX, and XI have been detected in RP patients, with type II collagen accounting for 95% of the total collagen content of cartilage and possibly representing the primary target of autoimmunity [1,4-6]. Additionally, genetic studies show HLA-DR4 to be a major risk allele for this disease [7]. The symptoms and clinical manifestations of RP vary based on the location of the involved cartilage, with auricular and nasal chondritis and polyarthritis being the most common. RP may also affect other proteoglycan-rich structures, including the eyes, heart valves, and blood vessels [1]. Most patients present between 40 and 60 years of age, but RP can occur at any age [1,8]. RP is associated with other autoimmune disorders in 30% of cases, more commonly rheumatoid arthritis [1], as was the case for our patient.

More recently, RP has been observed as a component of the newly defined VEXAS syndrome (Vacuoles, E1 enzyme, X-linked, Autoinflammatory, Somatic), which is an autoimmune inflammatory disorder caused by an acquired deficiency of the UBA1 gene in hematopoietic progenitor cells [9]. In a prospective observational cohort study, over 50% of VEXAS patients have been found to meet the diagnostic criteria for relapsing polychondritis [10]. Similarly, RP has shown associations with various bone marrow disorders, including hemolytic anemia, myelodysplastic syndrome, and myelodysplastic syndrome with hypoplasia or aplasia [11-13]. In fact, one in four cases of relapsing polychondritis is linked with myelodysplastic syndrome [12].

Our patient presented with auricular chondritis, the most common presentation of RP, which is observed in up to 90% of cases during the disease course and is the initial symptom in 20% of cases [1]. Auricular chondritis is characterized by painful erythematous to violaceous external ear inflammation, sparing the lobule [1,14]. Individual episodes resolve within days to weeks, but recurrent episodes of auricular chondritis can lead to a deformity known as “cauliflower ear” [8]. Other less common manifestations of relapsing auricular chondritis include temporary hearing loss and tinnitus [1]. Nasal chondritis may present as pain, erythema, and inflammation of the nose, with recurrent episodes ultimately leading to the destruction of the nasal bridge, known as “saddle-nose” deformity [8]. Symptoms such as cough, hoarseness, dyspnea, and wheezing can be seen in respiratory tract involvement and may signal potentially life-threatening airway collapse, the most common cause of death in RP [8].

The insidious onset, variable clinical manifestations, and overall poor knowledge of this rare condition by many clinicians make the diagnosis of RP challenging. Misdiagnosis and delayed diagnosis are common. A review by Trentham and Le found that the mean delay from the time medical attention was sought because of symptom onset until diagnosis was 2.9 years [15]. A retrospective cohort study of 87 patients by Zhang et al. found that 72% of cases of RP were misdiagnosed at the initial visits, all by non-rheumatologists. Laboratory findings may indicate inflammation or organ damage, but there are no specific diagnostic tests for RP, leaving the diagnosis to be made clinically using one of three accepted clinical criteria. Suggested diagnostic criteria established by McAdem et al. (1976) and modified by Damiani and Levine (1979) and Michet et al. (1986) are outlined in Table 1 [2,16,17]. Early, accurate diagnosis of RP and initiation of proper treatment are essential in preventing disease-related complications.

**Table 1:** Diagnostic criteria for relapsing polychondritis

References	Diagnostic Criteria
Mc Adam et al. 1976 [16]	At least 3/6 of the following symptoms: auricular chondritis, non-erosive seronegative inflammatory polyarthritis, nasal chondritis, ocular inflammation, respiratory tract chondritis, audiovestibular damage
Damiani and Levine 1979 [2]	3/6 of the McAdam’s criteria *OR* At least 1/6 symptoms in McAdam’s criteria plus a positive histological confirmation *OR* 2/6 symptoms in McAdam’s criteria plus positive response to corticosteroids or dapsone
Michet et al. 1986 [17]	Confirmed inflammation in 2/3 cartilages: auricular, nasal, or laryngotracheal *OR* Confirmed inflammation in 1/3 cartilages: auricular, nasal, or laryngotracheal, plus 2/3 minor criteria among hearing loss, ocular inflammation, vestibular dysfunction, seronegative arthritis

To date, there are no standardized guidelines on the treatment and management of RP. Management focuses on symptom resolution, cartilage preservation, and recurrence prevention. The choice of therapy is based on the clinical presentation, extent and severity of the disease, and site of involvement [14]. Non-steroidal anti-inflammatory drugs can treat mild clinical manifestations such as auricular or nasal chondritis. Dapsone and colchicine may also be considered for mild cases. More severe forms, including systemic vasculitis and ocular or laryngotracheal involvement, can be treated with systemic corticosteroids, immunosuppressant drugs, or biologics [14]. We found it surprising that our patient’s RP continued to flare despite receiving adalimumab for rheumatoid arthritis, which was well-controlled. To avoid adding a second systemic immunosuppressant, we chose to trial oral pentoxifylline 400 mg TID (off-label use) and topical clobetasol 0.05% ointment daily. Pentoxifylline is a phosphodiesterase inhibitor that increases cAMP and is primarily used in circulatory disorders. It increases red blood cell flexibility and decreases the viscosity of blood [18]. It is currently Food and Drug Administration (FDA)-approved for intermittent claudication, but it has been shown to have anti-inflammatory and immunomodulatory effects. It does so by inhibiting the production of inflammatory cytokines, depressing neutrophil degranulation, decreasing endothelial leukocyte adhesion, and lowering the sensitivity of leukocytes to cytokines [18]. Interestingly, pentoxifylline is used in veterinary medicine to treat vascular perfusion diseases of the pinnae (auricular cartilage) in cats and dogs, sharing overlapping pathogenic and clinical features with relapsing polychondritis [19]. Pentoxifylline has also been reported to show improvement in oral ulcers and arthritis in MAGIC (mouth and genital ulcers with inflamed cartilage) syndrome, likely attributed to its anti-inflammatory properties [20]. However, its potential benefit for chondritis was not commented on. Therefore, our literature search yielded no cases of RP being successfully treated with pentoxifylline in humans to date.

## Conclusions

Relapsing polychondritis is a rare, complex autoimmune disease with systemic complications that can lead to significant morbidity and mortality. Due to the rarity of the disease, variable clinical presentations (some without the more classic auricular or nasal chondritis), and recurrent episodic nature, RP poses a diagnostic challenge. Misdiagnosis and delayed diagnosis are common. Since RP is a clinical diagnosis and treatment is likely to involve multiple subspecialists, early recognition of this disease is critical. Additionally, a thorough understanding of its complex pathogenesis may have practical applications for treatment such as using pentoxifylline, as described in our case. Further research is needed to outline standardized guidelines on the diagnosis and treatment of relapsing polychondritis.
